# Cyclic AMP binding proteins in human breast cancer.

**DOI:** 10.1038/bjc.1985.224

**Published:** 1985-10

**Authors:** W. R. Miller, R. O. Senbanjo, J. Telford, D. M. Watson

## Abstract

The characteristics of a method for measuring cyclic AMP binding proteins in cytosols of human breast cancer are described. Using the assay, binding proteins were demonstrable in all of 100 tumour cytosols. Levels of binding in individual tumours varied from 0.8 to 15 pmol mg-1 cytosol protein (mean value 5 pmol mg-1 cytosol protein) and the dissociation constant ranged from 0.5 to 5.2 X 10(-8)M (mean 1.73 X 10(-8)M). Whilst replicate measurements within a single portion of tumour were reproducible (intra-assay coefficient of variation was between 4.5 and 7.8% and that for inter-assay variation was between 2.1 and 4.0%) there were often considerable differences in levels of binding proteins between different portions of the same tumour. Similar intra-tumour variations have been reported for other binding proteins and steroid receptors. The inter-relationships with such parameters may elucidate whether the differences are associated with variations in cellularity, cell type, or other specific factors.


*Br. J. Cancer* (1985), **52**, 531–535

### Cyclic AMP binding proteins in human breast cancer

W.R. Miller, R.O. Senbanjo, J. Telford & D.M.A. Watson

*University Department of Clinical Surgery, Royal Infirmary, Edinburgh EH3 9YW, Scotland, UK.*

**Summary** The characteristics of a method for measuring cyclic AMP binding proteins in cytosols of human breast cancer are described. Using the assay, binding proteins were demonstrable in all of 100 tumour cytosols. Levels of binding in individual tumours varied from 0.8 to 15 pmol mg$^{-1}$ cytosol protein (mean value 5 pmol mg$^{-1}$ cytosol protein) and the dissociation constant ranged from 0.5 to $5.2 \times 10^{-8}$ M (mean $1.73 \times 10^{-8}$ M). Whilst replicate measurements within a single portion of tumour were reproducible (intra-assay coefficient of variation was between 4.5 and 7.8% and that for inter-assay variation was between 2.1 and 4.0%) there were often considerable differences in levels of binding proteins between different portions of the same tumour. Similar intra-tumour variations have been reported for other binding proteins and steroid receptors. The inter-relationships with such parameters may elucidate whether the differences are associated with variations in cellularity, cell type, or other specific factors.

In experimental animals, cyclic AMP binding proteins are implicated in the growth of mammary tumours (Cho-Chung, *et al.*, 1978b; Bodwin, *et al.*, 1980; Bodwin *et al.*, 1981). The corresponding evidence in human breast cancers has yet to be fully documented. In the present paper we describe a method for measuring total cyclic AMP binding proteins in human breast tumour cytosols and some characteristics of the assay.

### Materials and methods

### *Reagents*

(5′8′-3H) Adenosine 3′,5′-cyclic phosphate, ammonium salt (45 Ci mmol) was obtained from Radiochemical Centre, Amersham, and radioinert adenosine 3′5′-cyclic phosphate, sodium salt from Sigma (Poole, UK). The following buffers were employed using analytical reagents – Buffer A 20 mM Tris, 0.25 M sucrose, 2 mM magnesium chloride, 1 mM calcium chloride, 10 mM potassium chloride, 16.26 mM HCl pH 7.5; Buffer B 55 mM potassium phosphate to which 11 mM theophylline was added immediately before use; Buffer C as Buffer B but with the addition of 10 mM magnesium chloride.

### *Tissues*

Breast cancers were obtained at mastectomy or biopsy from patients with histologically proven disease. All material was transported on ice to a cold room and processed immediately unless stated otherwise. The tumours represented 100 consecutive cases in which sufficient material was available for assay after tissue had been taken for routine histopathological examination and for oestrogen receptor analysis. Specimens were obtained from patients with T stage 1 to 4, although the number of T1 tumours was small.

### *Cytosol preparation*

All procedures were performed at 0–4°C. Tumour was dissected from surrounding fat and connective tissue, finely cut with scissors and homogenized in Buffer A (w/v 1:10) using a Silverson homogenizer at maximum speed for 20 sec then 15 sec, with 1 min interval for cooling. The homogenate was centrifuged at 105,000 *g* for 1 h in a MSE Superspeed 50 centrifuge and the resulting supernatant was used as cytosol.

### *Binding measurements*

Cytosol (50 μl) was incubated with 100 μl 5′,8′-[3H] cyclic AMP (25 nM to give a final concentration in the incubation system of 10 nM) and Buffer B (100 μl) containing radioinert cyclic AMP (final concentration 0,10,20,40,80, and 10,000 nM). Each system was set up in duplicate and incubated at room temperature for 3 h. To separate protein-bound cyclic AMP from free nucleotide, 2 ml Buffer B was added to each tube. The contents were then mixed and filtered through a Millipore filter (HAWP 0.45 μm) at 5 mm Hg negative pressure followed by 20 ml Buffer C at 10 mm Hg negative pressure. The filters were transferred to scintillation vials and dried under a stream of air. Micellar fluor NE 260, Nuclear Enterprises (5 ml) was added to each vial. The vials were then

Correspondence: W.R. Miller
Received 17 April 1985; and in revised form 11 June 1985.

© The Macmillan Press Ltd., 1985

incubated at 37°C for 2h and radioactivity was measured in a Tricarb liquid scintillation counter (Packard).

### *Cytosol protein*

The protein content of each cytosol was determined by the method of Bradford (1976) using bovine serum albumin as standard.

### Results

### *Assay conditions*

Tumour cytosols were incubated with [3H] cyclic AMP in the absence and presence of 10,000 nM radioinert cyclic AMP for varying times, either at 0°C or 20°C. A typical result is presented in Figure 1. Maximum binding at 20°C was achieved by 2h. Binding at 0°C was lower than at 20°C at each time point studied but was still increasing at 5h incubation. Overnight incubation at either 0°C or 20°C produced similar binding (data not shown). For routine assays it was decided to incubate at 20°C for 3h. The amount of binding under these conditions was linear with respect to increasing cytosol protein concentrations up to at least 3.0 mg ml$^{-1}$ (Figure 2). The effect of radioinert cyclic AMP on the binding of [3H] cyclic AMP is shown in Figure 3(a). Low concentrations of radioinert cyclic AMP were able to compete with [3H] cyclic AMP for binding, and there remained only a low level of non-specific binding in the presence of a thousand-fold excess of competitor. The data plotted according to Scatchard (1949), showed that the dissociation constant of binding was $2.7 \times 10^{-8}$ M and that the maximum concentration of binding sites within the assay system was about 2.0 nM (Figure 3b). Similar results were also obtained by performing the assay with increasing concentrations of radio-labelled ligand and assessing the non-specific binding by including a 100-fold excess of cold competitor at each of these concentrations (data not shown).

**Figure 1** The effect of time of incubation on the binding of [$^3$H] cyclic AMP to a cytosol of breast carcinoma either at 20°C (●) or 0°C (○). Each point represents the amount of [$^3$H] cyclic AMP bound in the absence of radioinert cyclic AMP corrected for that in the presence of 10,000 nM cold competitor. Remaining assay conditions as described in **Materials and methods.**

**Figure 2** The effect of cytosol concentration on the binding of [$^3$H] cyclic AMP to cytosols of 2 different breast carcinomas. Cytosols were prepared as described in **Materials and methods** and serially diluted to give the protein concentrations indicated. The diluted cytosols were incubated for 3h at 20°C with increasing concentrations of radioinert cyclic AMP. The data were analysed by Scatchard plot and each point represents the maximum number of binding sites for each system.

### *Values in breast cancer cytosols*

Cytosols from 100 primary breast cancers have been assayed for cyclic AMP binding proteins. The results are presented in Table I, and the concentration of binding sites in individual tumours are plotted in Figure 4. All tumours showed cyclic AMP binding but levels varied greatly between individual tumours, from 0.8 to 15 pmol mg$^{-1}$ cytosol protein.

**Table I** Levels and dissociation constants of cyclic AMP binding proteins in cytosols of 100 primary breast cancers

| | *Level* pmol mg$^{-1}$ *cytosol protein* | *Dissociation Constant* (M × $10^{-8}$) |
|---|---|---|
| Mean ± sd | 4.99 ± 3.07 | 1.72 ± 0.96 |
| Range | 0.80 – 15.05 | 0.5 – 5.2 |

**Figure 3** The effect of radioinert cyclic AMP on the binding of [$^3$H] cyclic AMP to a cytosol of human breast cancer. Assay conditions were as described in **Materials and methods**, data plotted as (a) radioactivity bound (b) according to Scatchard (1949).

**Figure 4** Levels of cyclic AMP binding proteins in cytosols of 100 primary breast cancers. Horizontal line represents mean value.

### *Reproducibility of measurements and effect of storage*

In order to determine the intra-assay precision of cyclic AMP binding measurements in tumours, large breast cancers were finely minced. Five portions, each of ~500 mg, were accurately weighed and cytosols were prepared separately and assayed. Two tumours were processed in this way; one possesed a mean value for cyclic AMP binding proteins from the 5 replicate estimations of 1.38 pmol mg$^{-1}$ cytosol protein with an intra-assay coefficient of variation of 7.9%, the other cancer had a mean value of 7.48 pmol mg$^{-1}$ cytosol protein with an intra-assay coefficient of variation of 4.5%.

To ascertain the interassay variation, 3 tumours were divided into 5 portions, as described for the study of intra-assay varitaion. One portion of each tumour was assayed for cyclic AMP binding proteins immediately (day 0) and the remaining portions were stored in separate vials in liquid nitrogen for 1,3,7 and 14 days until assayed. The results are shown in Figure 5. There appeared to be no observable decline in level of binding proteins with storage, and considering measurements within the same tumour as replicate estimates, the interassay coefficients of variation were 2.1%, 2.5% and 4.0% (that these values are lower than those for the intra-assay variation is probably a reflection of the larger number of simultaneous estimations performed in the study of intra-assay variation).

An estimate of the variation in cyclic AMP binding protein levels within individual cancers was obtained by dissecting out portions of tumours from central, intermediate and peripheral zones across each of 12 large breast cancers. These were assayed by the routine method and the results are presented in Figure 6 as ratios of the values relative to that in the peripheral zone. Whilst the mean of the 12 values found in each tumour zone were similar (and hence the mean value for the zone ratio was unity), there were often large variations in cyclic AMP binding protein levels between different

**Figure 5** The effect of storage in liquid nitrogen on reproducibility of cyclic AMP binding protein levels in human breast cancers. Three separate tumours were studied.

**Figure 6** Variation in cyclic AMP binding protein levels across 12 breast cancers. For experimental details, see text. Results have been expressed as a ratio of the value obtained from the intermediate area (I) or the central area (C) to that in the peripheral area (P).

areas in an individual tumour. This variation was invariably greater when comparing central and peripheral zones.

### Discussion

Measurements of cyclic AMP binding proteins in experimental animal cancers have yielded useful information regarding the state of automony of the tumours (Cho-Chung *et al.*, 1980*a*; Bodwin *et al.*, 1980). Similar data in human breast cancers has not yet been fully assessed. In the present paper we describe the characteristics of an assay which might be used routinely to measure total binding sites for cyclic AMP in cytosols of human breast cancers.

This method involves incubating tumour cytosol with radioactively labelled cyclic AMP in the absence and presence of increasing concentrations of radioinert competitor. At 20°C, maximum binding was achieved by 2 h and was linear between cytosol protein concentrations of 0.4 and 3 mg ml$^{-1}$. Under these conditions the non-specific binding assessed by adding 1000-fold excess of radioinert cyclic AMP was negligible ($<0.1\%$ of the added radioactivity). The binding capacity was not affected by storage up to 14 days in liquid nitrogen. The intra-assay coefficent of variation, as determined on aliquots from minced large tumours, was between 4.5 and 7.9%, and the inter-assay value was between 2.1 and 4.0%. These results are similar to those obtained by others using a different method (Kvinnsland *et al.*, 1983).

Using the present method, cytosols of 100 human breast cancers have been assayed for cyclic AMP binding. All possessed binding activity, levels varying from 0.8 to 15.0 pmol mg$^{-1}$ cytosol protein (mean value 5 pmol mg$^{-1}$ cytosol protein). These values fall within the range for human breast cancer cytosols reported by others using different methods (Eppenberger *et al.*, 1980; Kvinnsland *et al.*, 1983), and are also similar to those found in rat mammary tumours (Cho-Chung 1978*a*; Cho-Chung *et al.*, 1978*b*). The mean dissociation constant of $1.73 \times 10^{-8}$ M is also in keeping with data from experimental animal tumours (Cho-Chung *et al.*, 1978*b*).

It remains to determine which factors influence the levels of cyclic AMP binding proteins in cytosols of individual human breast cancers and, in particular, whether these levels are related to prognosis or endocrine responsiveness, as has been suggested by others (Kvinnsland *et al.*, 1983) and as is the case in rat mammary tumours (Cho-Chung 1978*a*, *b*, 1980). Assessments in breast cancers will have to take into account the variation in cyclic AMP binding proteins between different areas of the same tumour. Data from the present study shows that there may be considerable differences in the level of cyclic AMP binding between each of three different areas (central, peripheral and intermediate) of large tumours. No consistent pattern of variation across the tumours was evident and the *mean* value for cyclic AMP binding in this group of cancers was similar, irrespective of the area of tumour upon which the estimation was performed. At present, it is not known whether these differences within tumours are associated with variations in cellularity, cell type or other factors. Similar intra-tumour variations have been noted with other binding proteins such as the oestrogen receptor (Hawkins *et al.*, 1977; Silversward *et al.*, 1980) and the inter-relationship with these different types of binding protein may help to elucidate the problem.

The authors thank Professor A.P.M. Forrest for allowing them to study material from patients under his care and for the interest he has shown in the work and Dr R.A. Hawkins for his helpful suggestions and comments. We also gratefully acknowledge the support of the Medical Research Council (Grant No G 979/693/CA).

### References

BODWIN, J.S., CLAIR, T., & CHO-CHUNG, Y.S. (1980). Relationship of hormone dependency to oestrogen receptor and adenosine 3′,5′-cyclic monophosphate-binding proteins in rat mammary tumours. *J. Natl Cancer Inst.*, **64**, 395.

BODWIN, J.S., HIRAYAMA, P.H., REGO, J.A. & CHO-CHUNG, Y.S. (1981). Regression of hormone-dependent mammary tumours in Sprague-Dawley rats as a result of tamoxifen or pharmacologic doses of estradiol: cyclic adenosine 3′,5′-monophosphate-mediated events. *J. Natl Cancer Inst.*, **66**, 321.

BRADFORD, M.M., (1976). A rapid and sensitive method for the quantitation of microgram quantities of protein utilizing the principle of protein-dye binding. *Anal. Biochem.*, **72**, 248.

CHO-CHUNG, Y.S., (1978*a*). Antagonistic action between cyclic adenosine 3′,5′-monophosphate and oestrogen in rat mammary tumour growth control. *Cancer Res.*, **38**, 4071.

CHO-CHUNG, Y.S., (1980*a*). Cyclic AMP and its receptor protein in tumour growth regulation *in vivo*. *J. Cyclic Nucleotide Res.*, **6**, 163.

CHO-CHUNG, Y.S., BODWIN, J.S. & CLAIR, T. (1978*b*). Cyclic AMP binding proteins. Inverse relationship with oestrogen receptors in hormone dependent tumour regression. *Europ. J. Biochem.*, **86**, 51.

CHO-CHUNG, Y.S., CLAIR, T., SCHWIMMER, M., STEINBERG, L., REGO, J. & GRANTHAM, F. (1980*b*). Cyclic adenosine 3',5'-monophosphate receptor proteins in hormone-dependent and -independent rat mammary tumours. *Cancer Res.*, **41**, 1840

EPPENBERGER, U., BIEDERMANN, K., HANDSCHIN, J.C., FABBRO, D., KUNG, W., HUBER, P.R. & ROOS, W. (1980). Cyclic AMP-dependent protein kinase type I and type II and cyclic AMP-binding in human mammary tumours. *Adv. Cyclic Nucleotide Res.*, **12**, 123,

HAWKINS, R.A., HILL, A., FREEDMAN, B., GORE, S.M., ROBERTS, M.M., & FORREST, A.P.M. (1977). Reproducibility of measurements of oestrogen receptor concentration in breast cancer. *Br. J. Cancer*, **36**, 355.

KVINNSLAND, S., EKANGER, R., DOSKELAND, S.O. & THORSEN, T. (1983). Relationship of cyclic AMP binding capacity and oestrogen receptor to hormone sensitivity in human breast cancer. *Breast Cancer Res. & Treatment*, **3**, 67.

SCATCHARD, G. (1949). The attraction of proteins for small molecules and ions. *Ann. N.Y. Acad. Sci.*, **51**, 660.

SILVERSWARD, C., SKOORL, G., HUMLA, S., GUSTAFSSON, S.A. & NORDENSKJOLD, B. (1980). Intra tumoural variation of cytoplasmic and nuclear oestrogen receptor concentrations in human mammary caricinoma. *Europ. J. Cancer*, **16**, 59.


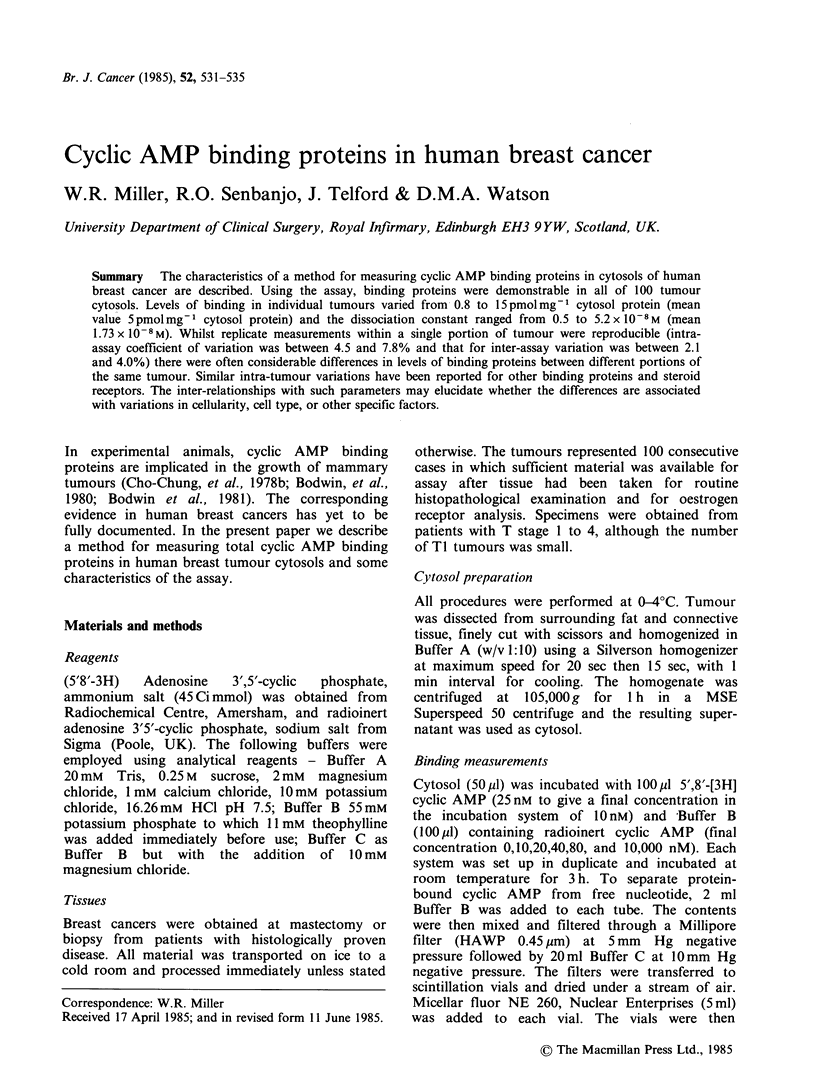

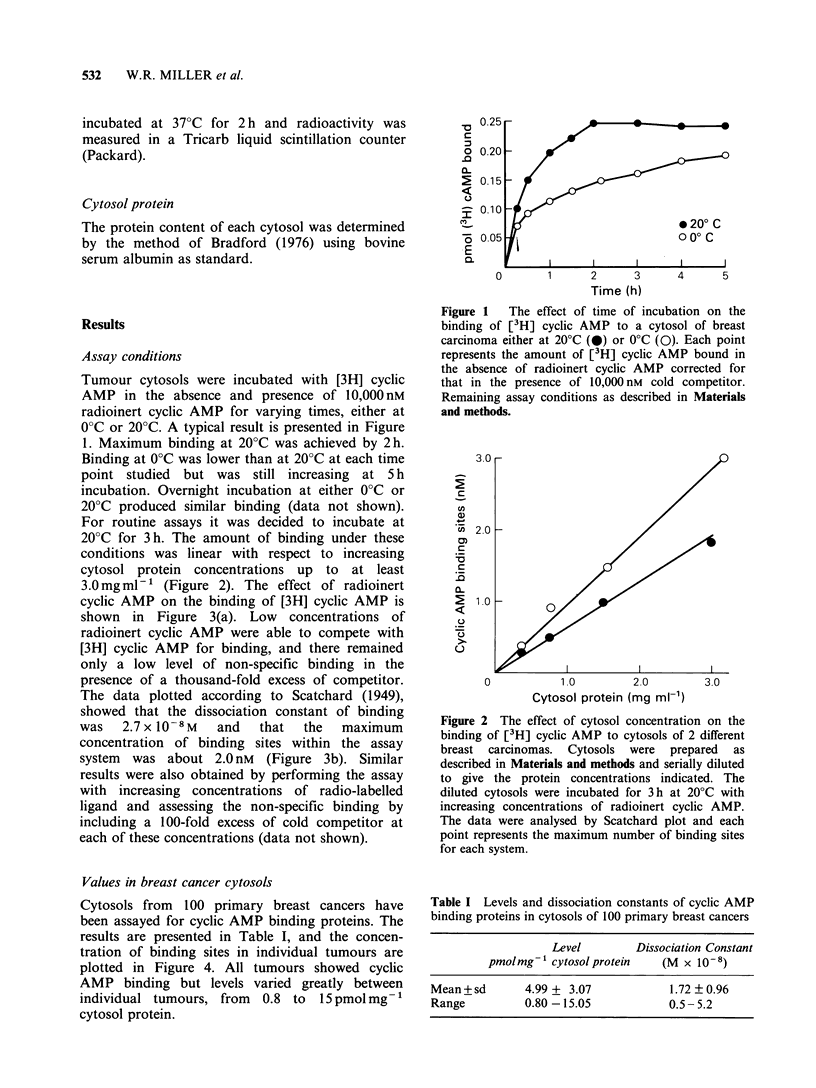

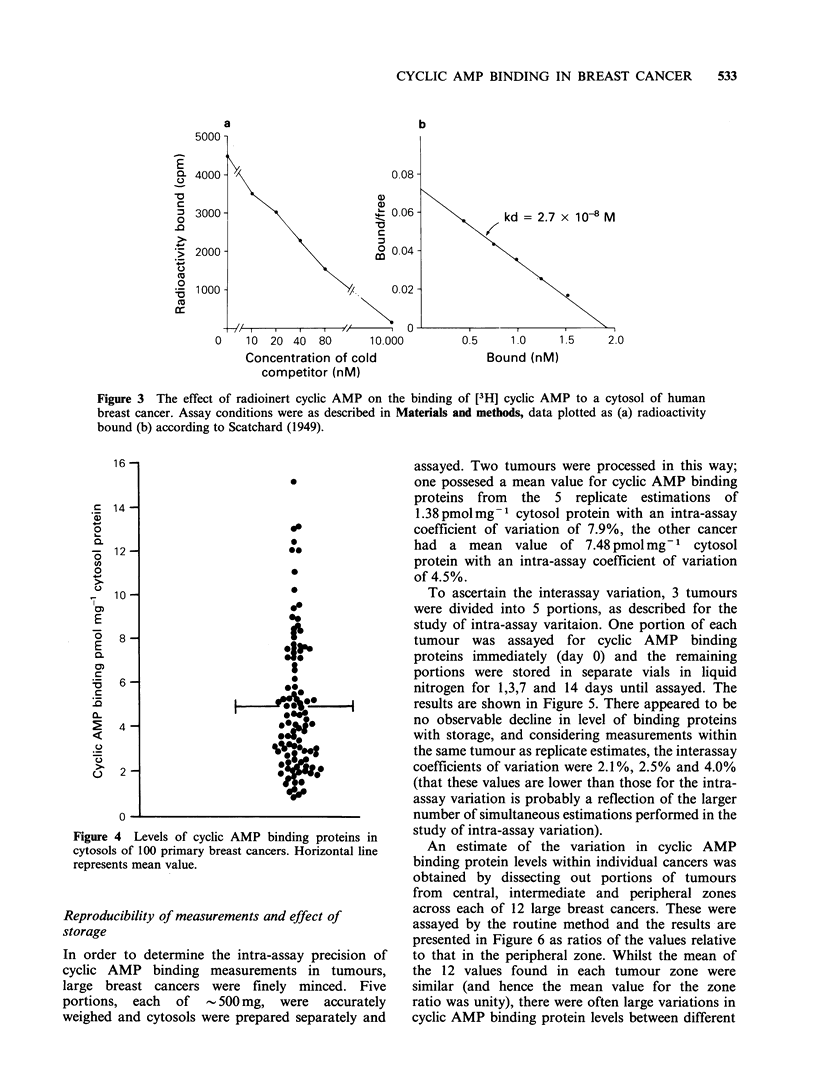

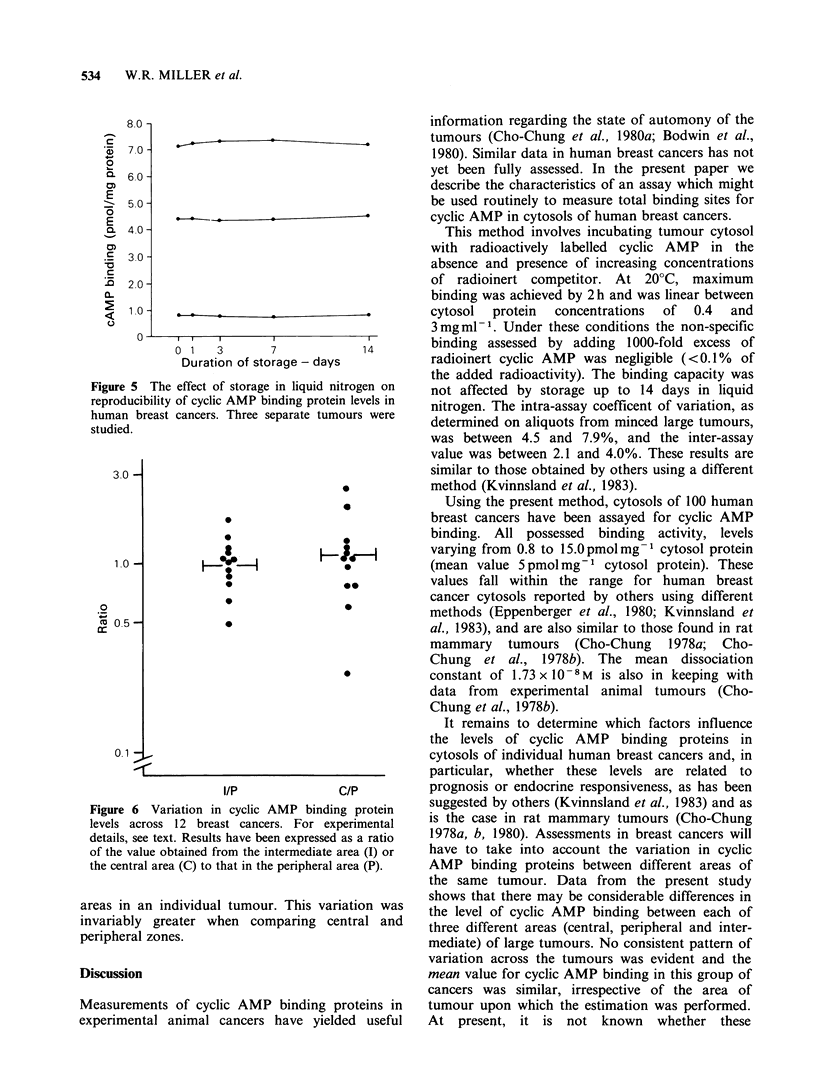

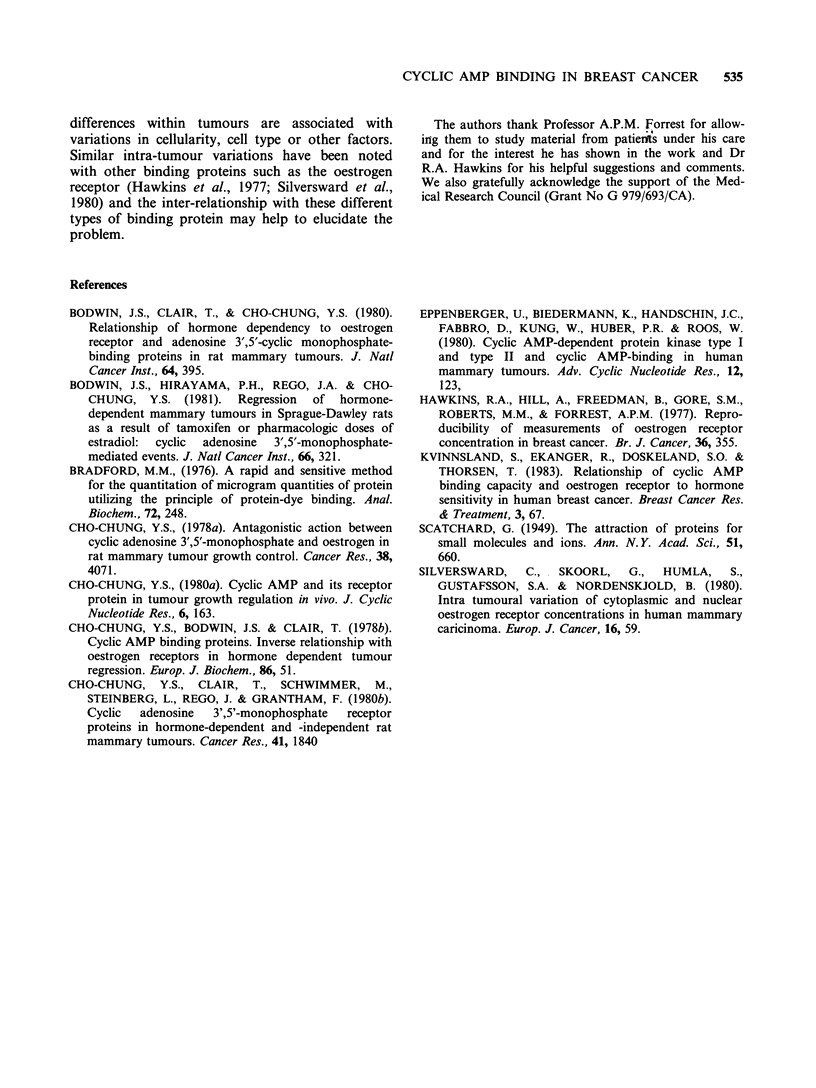


## References

[OCR_00566] Bodwin J. S., Clair T., Cho-Chung Y. S. (1980). Relationship of hormone dependency to estrogen receptor and adenosine 3',5'-cyclic monophosphate-binding proteins in rat mammary tumors.. J Natl Cancer Inst.

[OCR_00575] Bodwin J. S., Hirayama P. H., Rego J. A., Cho-Chung Y. S. (1981). Regression of hormone-dependent mammary tumors in Sprague-Dawley rats as a result of tamoxifen or pharmacologic doses of 17 beta-estradiol: cyclic adenosine 3',5'-monophosphate-mediated events.. J Natl Cancer Inst.

[OCR_00583] Bradford M. M. (1976). A rapid and sensitive method for the quantitation of microgram quantities of protein utilizing the principle of protein-dye binding.. Anal Biochem.

[OCR_00587] Cho-Chung Y. S. (1978). Antagonistic action between cyclic adenosine 3':5'-monophosphate and estrogen in rat mammary tumor growth control.. Cancer Res.

[OCR_00598] Cho-Chung Y. S., Bodwin J. S., Clair T. (1978). Cyclic AMP-binding proteins: inverse relationship with estrogen-receptors in hormone-dependent mammary tumor regression.. Eur J Biochem.

[OCR_00604] Cho-Chung Y. S., Clair T., Schwimmer M., Steinberg L., Rego J., Grantham F. (1981). Cyclic adenosine 3':5-monophosphate receptor proteins in hormone-dependent and -independent rat mammary tumors.. Cancer Res.

[OCR_00593] Cho-Chung Y. S. (1980). Hypothesis. Cyclic AMP and its receptor protein in tumor growth regulation in vivo.. J Cyclic Nucleotide Res.

[OCR_00611] Eppenberger U., Biedermann K., Handschin J. C., Fabbro D., Küng W., Huber P. R., Roos W. (1980). Cyclic AMP-dependent protein kinase type I and type II and cyclic AMP binding in human mammary tumors.. Adv Cyclic Nucleotide Res.

[OCR_00619] Hawkins R. A., Hill A., Freedman B., Gore S. M., Roberts M. M., Forrest A. P. (1977). Reproducibility of measurements of oestrogen-receptor concentration in breast cancer.. Br J Cancer.

[OCR_00625] Kvinnsland S., Ekanger R., Døskeland S. O., Thorsen T. (1983). Relationship of cyclic AMP binding capacity and estrogen receptor to hormone sensitivity in human breast cancer.. Breast Cancer Res Treat.

[OCR_00637] Silfverswärd C., Skoog L., Humla S., Gustafsson S. A., Nordenskjöld B. (1980). Intratumoral variation of cytoplasmic and nuclear estrogen receptor concentrations in human mammary carcinoma.. Eur J Cancer.

